# Practical Challenges of DBT-Guided VABB: Harms and Benefits, from Literature to Clinical Experience

**DOI:** 10.3390/cancers15245720

**Published:** 2023-12-06

**Authors:** Daniele Ugo Tari, Davide Raffaele De Lucia, Marika Santarsiere, Rosalinda Santonastaso, Fabio Pinto

**Affiliations:** 1 Department of Breast Imaging, Caserta Local Health Authority, District 12 “Palazzo della Salute”, 81100 Caserta, Italy; dav.delucia@gmail.com (D.R.D.L.); marika.santarsiere@gmail.com (M.S.); 2 Department of Economics, University of Campania “L. Vanvitelli”, 81043 Capua, Italy; rosalinda.santonastaso@unicampania.it; 3 Department of Radiology, “A. Guerriero” Hospital, Caserta Local Health Authority, 81025 Marcianise, Italy; fpinto1966@libero.it

**Keywords:** breast cancer, screening, digital breast tomosynthesis, vacuum-assisted breast biopsy, breast biopsy, breast density, mammography

## Abstract

**Simple Summary:**

Vacuum-assisted breast biopsy (VABB) represents one of the best instruments for radiologists to obtain a histological diagnosis of suspicious lesions on imaging. The advent of digital breast tomosynthesis (DBT)-guided VABB has increased the accuracy of diagnosis in the most complex lesions, particularly those without ultrasound correlation or those visible only on DBT. In this paper, we retrospectively analyzed our experience with VABB. Starting from a review of the literature, we evaluated the challenges arising from clinical practice to provide useful indications for the implementation of this technique. We identified 37 breast cancers at an early stage with a low complication rate and high histopathological concordance between VABB and surgery. Compared to the literature, our results confirmed the fundamental role of DBT-VABB in achieving a timely diagnosis for nonpalpable lesions, offering a safe and minimally invasive approach when the technique is executed correctly.

**Abstract:**

Vacuum-assisted breast biopsy (VABB) guided by digital breast tomosynthesis (DBT) represents one of the best instruments to obtain a histological diagnosis of suspicious lesions with no ultrasound correlation or those which are visible only on DBT. After a review of the literature, we retrospectively analyzed the DBT-guided VABBs performed from 2019 to 2022 at our department. Descriptive statistics, Pearson’s correlation and χ^2^ test were used to compare distributions of age, breast density (BD) and early performance measures including histopathology. We used kappa statistics to evaluate the agreement between histological assessment and diagnosis. Finally, we compared our experience to the literature to provide indications for clinical practice. We included 85 women aged 41–84 years old. We identified 37 breast cancers (BC), 26 stage 0 and 11 stage IA. 67.5% of BC was diagnosed in women with high BD. The agreement between VABB and surgery was 0.92 (k value, 95% CI: 0.76–1.08). We found a statistically significant inverse correlation between age and BD. The post-procedural clip was correctly positioned in 88.2%. The post-procedural hematoma rate was 14.1%. No infection or hemorrhage were recorded. When executed correctly, DBT-guided VABB represents a safe and minimally invasive technique with high histopathological concordance, for detecting nonpalpable lesions without ultrasound correlation.

## 1. Introduction

Breast cancer (BC) is the most frequent malignancy in the female population in terms of incidence and mortality, accounting for 2,261,419 (11.7%) of new cases and 684,996 (6.9%) of deaths for cancer worldwide [1]. Early diagnosis is universally recognized as the best way to face this disease and biennial screening for women at average risk for breast cancer aged 50 to 69 years in well-resourced settings is recommended by the World Health Organization (WHO) [2,3].

Conventional two-dimensional (2D) full-field digital mammography (FFDM) is considered the gold standard for early diagnosis in BC screening programs, but the potential overlap of tissue in dense breasts is a major limitation, because it can obscure an area of interest and lead to a false-negative finding [4]. In addition, the overlap of normal structures can create a pseudolesion, often called summation artifact, which drives to a false-positive result [5]. Digital breast tomosynthesis (DBT), based on the acquisition of a series of low-dose projection images, diminishes the masking effect of tissue overlap and structure noise usually found in 2D-FFDM, increasing cancer detection rates, and decreasing false-positive rates [6,7].

Nowadays, for the diagnosis of nonpalpable image-detected abnormalities, according to triple test, which means that all the findings of cytology and histology should be correlated with the radiological assessment, core needle biopsy (CNB) is a widely accepted tissue-sampling method alternative to open excision biopsies [8]. Another technique currently used for breast biopsies is the vacuum-assisted breast biopsy (VABB).

VABB was developed in 1995 by Fred Burbank and Mark Retchard to overcome the shortcomings of core biopsies, such as insufficient amount of tissue for diagnosis or incorrect targeted of non-palpable lesions [9]. The VABB needles are more accurate and allow larger volume tissue sampling than automated biopsy needles. Furthermore, this procedure provides a higher calcification retrieval rate and a lower rate of targeting errors in comparison to CNB, decreasing re-biopsy and underestimation rates. The needle size ranges from 7 to 12 gauge (G). Depending on the type of lesion and size of the biopsy needle, on average, at least 6–12 samples are obtained [10,11]. It is possible to perform VABB with ultrasound (US), stereotactic and/or DBT, and magnetic resonance imaging (MRI) guidance.

Stereotactic and/or DBT-guided breast biopsy is the method of choice for lesions detected only on mammography, including microcalcifications, masses, asymmetries, and architectural distortions. For lesions seen only or more clearly on DBT than on 2D-FFDM, DBT-guided percutaneous biopsy is preferred if available [12]. Most common indications for stereotactic and/or DBT-guided breast biopsies are microcalcifications and noncalcified lesions, such as architectural distortions (AD), focal asymmetries or asymmetries, seen on one view only or without US correlate; re-biopsy of discordant cases or clarification of some lesions, such as atypical ductal hyperplasia (ADH), atypical lobular hyperplasia (ALH), lobular carcinoma in situ (LCIS), papillary lesions, radial scar, flat epithelial atypia (FEA); multiple suspicious lesions, particularly in a multifocal or multicentric distribution, to facilitate treatment planning; lesions seen on mammography that correlate with suspicious areas of enhancement present on contrast-enhanced breast MRI [13]. According to Breast Imaging Reporting and Data System (BI-RADS^®^), all these lesions can be classified as probably benign (R3 on mammography, U3 on ultrasound), suspicious (R4, U4) or highly suggestive of malignancy (R5, U5). BI-RADS^®^ Category 4 can be further subdivided in 4a, 4b and 4c, depending on the degree of suspicion—low, moderate and high, respectively [14].

Unlike 2D stereotactic VABB, performed usually with patient in prone position, the DBT-guided VABB may be performed in both upright position on a routinely used tomosynthesis machine and prone on a table with tomosynthesis biopsy capability.

At our department, we perform upright DBT-guided biopsies while a prone table is forthcoming. In this paper, we aimed to present our experience with VABB. Starting from a review of the current state of the art, we analyzed the challenges arising from clinical practice, in order to provide useful indications and practical advice for the implementation of the technique.

## 2. Materials and Methods

### 2.1. Study Design

We performed a retrospective study following the STROBE guidelines (Table S1). We retrospectively analyzed the DBT-guided VABB procedures performed from 2019 to 2022 at our department. We exclusively enrolled female patients aged 40 and above, who underwent screening mammography in double projection (medio-lateral-oblique, MLO; cranio-caudal, CC) at our department and had a well-documented clinical history. Women with a pre-existing diagnosis of breast cancer and/or those who did not undergo the preliminary examination at our institution were excluded from the study. All patients with doubtful or positive findings at VABB (>B3) underwent surgery. For benign findings, we considered the available histopathology of the VABB or of the surgery, if performed.

At our department, screening mammography is biennial for women aged between 50 and 69 years old, and annual for women aged >40 years with a familiar history of breast cancer. We performed DBT in double projection with 2D synthetic reconstruction (2Ds), in double reading with a further evaluation by a third radiologist in discordant cases. Clinical history is recorded in Caserta Local Health Authority’s online web portal named Sani.ARP.

During the COVID-19 pandemic, our activities were reduced due to emergency, but we still performed several procedures according to the available guidelines [15].

### 2.2. Statistical Analysis

Descriptive statistics were used to evaluate the distributions of age, breast density and early performance measures, including histopathologic tumor characteristics. Age is presented using median, means and standard deviation (SD). Therefore, we calculated these parameters for the whole sample and for each subgroup of findings, including breast density and diagnoses. A Pearson’s correlation has been performed to evaluate the number of breast cancers diagnosed in relation to age and breast density. A χ^2^ test has been performed to compare breast density categories and diagnosis. The agreement between histological assessment after VABB and diagnosis after surgery was assessed with weighted kappa statistics. A *p* < 0.05 was considered statistically significant, with a 95% confidence interval. According to Landis J.R. and Koch G.G., the kappa values were interpreted as follows: 0.0–0.20, slight agreement; 0.21–0.40, fair agreement; 0.41–0.60, moderate agreement; 0.61–0.80, substantial agreement; 0.81–1.00, almost perfect agreement [4].

Statistical analyses were performed by using STATA 13 (StataCorp LLC, College Station, TX, USA).

Breast density was evaluated with Quantra software version 2.2.3 (Hologic, Bedford, MA, USA) or, when not available, by visual assessment of radiologists according to BIRADS 5th Edition [14], where category A indicates almost entirely fat; category B shows scattered areas of fibroglandular tissue; category C represents heterogeneously dense that may obscure small masses; category D indicates extremely dense, lowering the sensitivity of the mammography. BI-RADS assessment categories A and B were considered non-dense, and categories C and D were considered dense. The histopathological B classification was defined according to guidelines for non-operative diagnostic procedures and reporting in breast cancer screening [16]: B1, normal tissue; B2, benign lesion; B3, lesion of uncertain malignant potential; B4, suspicious; B5, malignant.

Finally, starting from a review of the different available VABB techniques, we compared our experience with what is reported in the literature, analyzing the differences and similarities in terms of indications, approach, results and complications. In conclusion, we provided practical advice for clinical practice.

### 2.3. VABB Procedures with Upright System

**Figure 1:**
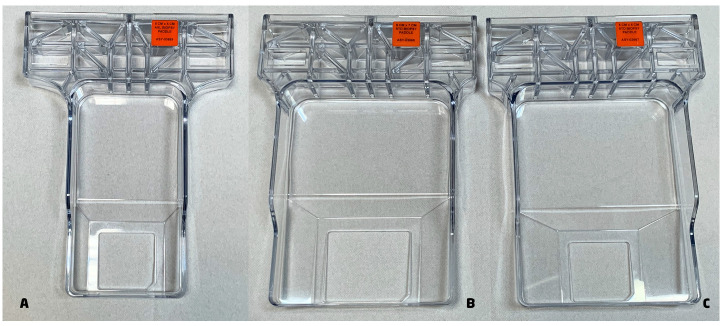
Paddles. Three types of compression paddles that can be used to perform VABB: (**A**) 5 × 5 cm axillary biopsy paddle; (**B**) 6 × 7 cm standard biopsy paddle; (**C**) 5 × 5 cm standard biopsy paddle.

Upright DBT-guided biopsies are performed on a digital mammography system with a 3D tomosynthesis platform by using an installed guidance system. The full detector is used with varying compression paddles (small, large, and axillary) (Figure 1).

We used a dedicated armchair that allows various positioning options. Specifically, the patients are sitting upright when a superior, medial, or lateral approach is used and in the lateral decubitus position for an inferior approach. This is due to the limitation that the C-arm and gantry cannot be rotated a full 180° to have the biopsy window open inferiorly.

The most correct approach must be carefully chosen in the planning phase in agreement with the team in charge. The decision is based on the view in which the lesion is best seen, also considering the shortest distance from the skin to the lesion and avoidance of intervening vessels, the patient’s breast size, compressed breast thickness, and lesion’s depth and location. Then the breast is fixed and compressed and a DBT is performed to identify the target lesion.

**Figure 2:**
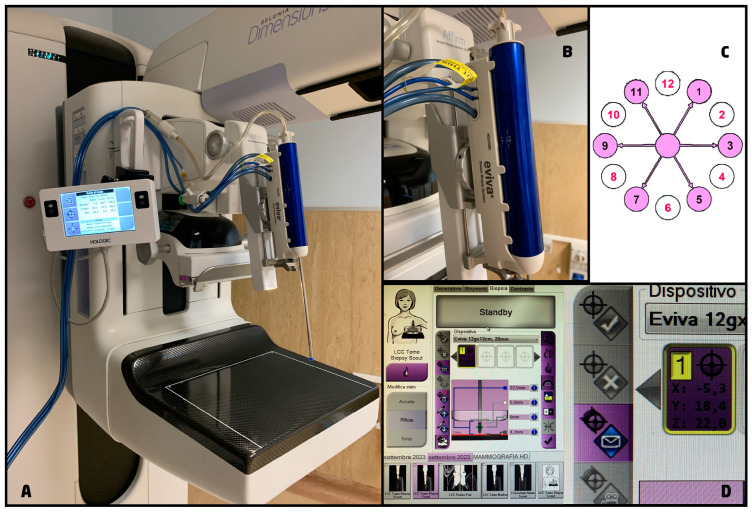
Biopsy system. (**A**) Affirm^®^ Upright Breast Biopsy Guidance System (Hologic, Bedford, MA, USA), it added to the mammography unit to perform 2D or DBT-guided biopsies and it is equipped with a monitor to display the biopsy coordinates and the needle position; (**B**) Eviva ^®^ breast biopsy system (Hologic, Bedford, MA, USA), it is available with different needles options and makes possible saline lavage and constant aspiration thanks to its connection to the vacuum console; (**C**) scheme showing the range of movements of the biopsy system for tissue sampling at even (white circles) and odds (pink circles) positions; (**D**) an example of how to calculate the coordinate for the biopsy using a 12 gauge needle.

After the radiologist has selected the lesion on the acquired DBT images, by identifying the DBT section that yielded the sharpest depiction of the target, the biopsy coordinates are automatically calculated by the system, including z-axis location (Figure 2A–D).

After the skin disinfection, local anesthesia is administered. The biopsy needle is inserted in the parenchyma according to the calculated coordinates and pre-fire stereotactic images are taken to confirm the expected needle trajectory. Postfire stereotactic images, with a needle placed through the target lesion, are acquired at the discretion of the radiologist. Then the tissue sampling in a 360° fashion is performed. Considering that the opening of the sampling chamber is by convention directed towards the chest wall, at 12 o’clock, regardless of the approach, tissue sampling may be performed allowing an average of 6 to 12 samples, generally taken at even or odd positions of the quadrant, rotating 360° (Figure 2C). Pre- and post-fire control images are usually acquired as stereotactic FFDM images because the inserted needle would lead to artifacts at DBT. A localizing post-biopsy marker clip, usually a 3 mm titanium clip, is then placed at the biopsy site, to future mammographic monitoring of the area or to guide surgical excision. After the needle is removed and hemostasis is achieved, a two-view FFDM is obtained to confirm that the target lesion is removed and the biopsy marking clip has been released. If necessary, a specimen radiograph is then obtained to confirm the presence targeted lesion, such as microcalcifications [12,17,18].

### 2.4. VABB Procedures with Prone System

Biopsies performed in prone position use dedicated biopsy tables. The patient must be able to get on the table and lie prone for approximately 20–30 min with the targeted breast pendant through an aperture in the table. A weight limit of the patient, generally between 136 and 158 kg, should be considered because exceeding the limit on the table may cause mechanical failure [11]. As previously mentioned, also in this case, the patient is positioned basing on the shortest and most accessible distance and route to the target lesion.

In prone stereotactic (PS) VABB, after the compression, a scout image is acquired to identify the target lesion. Once the target is confirmed, stereotactic pair images at +15° and −15° are obtained calculating the coordinates of the lesion [19].

Prone DBT-VABB procedures are performed with a dedicated system with tomosynthesis-guided biopsy capability, which allows for placing the patient on both sides of the table. It is generally equipped with an independently rotating biopsy arm, permitting a 360° approach to the breast [20]. After positioning, the procedure continues in the same way as the upright technique.

### 2.5. Side Effects and Troubleshooting

#### 2.5.1. Side Effects

VABB is a safe minimal invasive procedure, but side effects may occur, and the team needs to be trained in recognizing the signs and in their management. The most common side effects include:

- Vasovagal reaction (0.3%). It manifests as flushing, nausea, feelings of lightheadedness and diaphoresis. Its management usually involves removal of the needle if already positioned while checking the patient’s vital signs, decompression of the breast, then lying the patient down possibly by elevating the lower limbs, applying a cool washcloth to the forehead and neck and ventilating. [21,22]. It is also good practice to consider offering sugary drinks such as fruit juices or soft drinks to quickly combat low blood sugar, especially in case of patients with diabetes [23].
- Bleeding. The risk of significant bleeding hemorrhage is about 0.1% [21]. It can manage with manual compression for about 5–10 min and bandaging which are routinely implemented to reduce the extent of post-procedural bleeding. In particular, in cases of more prolonged bleeding, an elastic compression bandage and a cold ice pack may be useful, as well as the administration of vitamin K, either orally, sublingually or directly at the bleeding site may help reduce the extent of bleeding and stop it. Furthermore, the use of lidocaine with epinephrine for deep anesthesia and biopsy cavity lavage can reduce bleeding [23]. After biopsy, most patients present with superficial ecchymosis due to minor bleeding. In addition, the development of a postprocedural hematoma is a further possible complication, the incidence and severity of which are directly related to the gauge of the needle used. Schaefer et al. [24] classified hematomas as small (within 15 mm), moderate (between 15 and 30 mm) and severe (between 30 and 40 mm). In their experience, they found a rate of small and moderate/severe hematomas of 29% and 12.9%, respectively, when using 8G/9G needles, and of 6% and 2.4%, respectively, when using 11G/12G needles.
- Infection (<1%). It should be limited by avoiding cross-contamination of clean (e.g., mammography unit, DBT equipment) and sterile (e.g., skin, biopsy needle, tissue marker) surfaces by operators as much as possible, using sterile gloves and disinfecting the skin area of the breast subject to collection. If mastitis develops after biopsy, it can usually be managed with oral medications on an outpatient basis [21].

#### 2.5.2. Comorbidity

The pre-existence of mental, psycho-social, or physical comorbidities may limit patients’ tolerability of the procedure. In case of anxiety problems, the use of anxiolytic drugs should be considered prior to the procedure. For patients with mental problems or dementia, having a family member or carer in the room may be helpful during the biopsy to help explain or follow directions. For patients with disabilities or physical limitations, a different position or the use of an alternative method should be considered, either ultrasound-guided, which is quicker and more tolerated, or directly surgical excision, although in any case an upright operating unit is usually more suitable for the patient [25].

#### 2.5.3. Patients on Anticoagulant Therapy

At the planning stage, it is essential to collect the patient’s clinical and pharmacological history, in particular by determining whether there is a history of hemorrhagic diathesis and/or an ongoing anticoagulant or antiplatelet drugs therapy [26].

According to the sources found in the literature, anticoagulant drugs such as aspirin, warfarin, non-steroidal anti-inflammatory drugs (NSAIDs) and clopidogrel should not be routinely discontinued prior to image-guided breast biopsy [27,28,29]. If considering the type of biopsy and of the needles used (a 14-gauge automatic needle or a 9–14-gauge vacuum needle), there is no statistically significant difference in hematoma formation when comparing patients taking anticoagulant drugs with those not taking anticoagulant drugs [26,27]; however, a significant increase in bruising may occur in patients receiving anticoagulant therapy [26].

Good clinical practice involves the use of the Society of Interventional Radiology (SIR) recommendations on anticoagulation management for core needle biopsy, where breast biopsies are at low risk for bleeding and do not require a change in anticoagulant medication in most cases [30]. Nevertheless, the management of patients should always be personalized and thus the discontinuation or not of anticoagulants is based on the patient’s risk of bleeding and thromboembolism, as well as her clinical status. Consequently, laboratory tests such as prothrombin time (PT), international normalized ratio (INR) and platelet count analysis should be routinely recommended, especially in case of warfarin therapy. Moreover, for patients with a complex medical history, the cardiologist, hematologist and/or neurologist should be consulted before changing drug therapy [30].

#### 2.5.4. Difficult Targets

As previously mentioned, the most recent VABB systems allow semi-automatic identification of the target. Nevertheless, it is possible to face targeting problems commonly caused by the following conditions:

- Thin breasts. A breast thickness of less than 2 cm can make biopsy difficult. The use of a smaller needle, with smaller aperture, minimizes the risk of posterior skin and/or support plate compromise. Other methods to address this problem include reducing breast compression, building up breast tissue by using towels or gauze against the posterior imaging plate, compression by generating anterior bulging effect, or creating a breast sling by pulling the breast tissue upward and taping the underside when the biopsy is performed in the lateral approach, and finally, the use of lidocaine to develop a wheel at the biopsy entry site [25,31,32].
- Vessels. The use of DBT is useful to identify and avoid vessels along the needle path. Vessels at least 15 mm deep relative to the target may not be a problem. In other cases, an alternative approach may be considered [25].
- Surface targets. A lesion close to the skin presents a unique challenge as the biopsy furrow may not be completely covered by the skin. When the skin is exposed to the groove, the vacuum may pull the skin, resulting in a much larger skin slit and an increased risk of infection [25]. Some steps that can be taken to avoid this problem include choosing a small needle with a smaller sulcus, using a skin hook to pull the skin over the exposed sulcus, and advancing the needle into the breast so that the target is positioned toward the proximal end of the sulcus instead of in the center [25,31,32].
- Posterior targeting. The attachment of posterior lesions is usually facilitated using the upright system, but in any case, lesions close to the chest wall should be targeted in the anterior portion by then trying to take more material toward the chest wall. A mediolateral oblique (MLO) position will allow more posterior and medial tissue to be visualized [31].
- Droopy breasts. Larger breasts can result in slippage effects of the tissues underlying the skin, or displacement of the breast as a result of insufficient traction. This drawback can be recognized in pre-fire images when the target is shown below the needle. In these cases, it is necessary to remove the needle, dry the breast, and perform another attempt using the same approach with increased compression. Alternatively, the approach can be changed to cranio-caudal (CC) projection if possible [25].
- Defective marker release. At the end of the procedure, after releasing the marker, a post-biopsy image with the breast still compressed and the sheath still in place should be taken to confirm correct placement. If no marker is seen, another biopsy marker should be placed. In cases of superficial biopsy or significant bleeding, a biopsy marker may extrude from the breast after the breast has been released from compression [33]. In this situation, it is suggested to put the breast back into compression immediately to place another marker or to place it 2–3 days after the procedure at about 5–10 mm proximal to the end of the hematoma, where the center of the biopsy cavity is expected to be [25].

#### 2.5.5. Biopsy Cancellation

Biopsy cancellation rate ranges from 2% to 13%, with a rate of malignancy founded at re-biopsy of 18–58% [25,33,34]. Failure to identify the lesion at the time of biopsy is the most frequent reason for cancellation followed by the patient’s failure to tolerate the procedure. Other reasons include lesion location, proximity to the chest wall, and thin breast [34,35,36]. In cases of voiding, the target should undergo different management according to the level of suspicion. Indeed, a surgical excision should be considered in cases of strong suspicion on imaging; otherwise, a supplementation with contrast medium MRI or contrast-enhanced digital mammography (CEDM) imaging method may be useful as well as a short follow-up with mammography for possible early detection of malignancy. In a study by Jackman and Marzoni [36] of 42 lesions in which stereotactic biopsy was cancelled and the lesion was downgraded to BI-RADS category 2 or 3, malignancy was found in 5% of the lesions.

### 2.6. VABB Procedure at Our Department

The patient preparation at our department consists in a brief description of the upcoming procedure and the accurate analysis of the patient medical history and of the requested laboratory tests such as prothrombin time (PT), partial thromboplastin time (PTT), international normalized ratio (INR), and platelet count analysis. Finally, the written informed consent form, including a description of the procedure and the associated risks and benefits, is signed.

**Figure 3:**
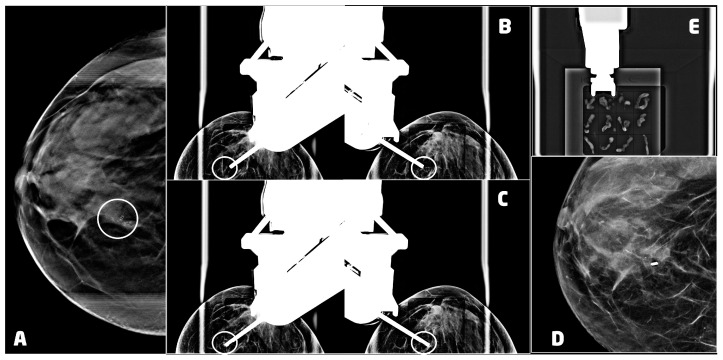
VABB upright procedure for a 53 years old women with a ductal carcinoma in situ, G3 of the right breast: (**A**) digital breast tomosynthesis of the right breast, in cranio-caudal projection, showing a cluster of microcalcifications at the union of the inner quadrants (circle); (**B**) pre-fire stereotactic images of the right breast showing the position of the needle near the suspicious lesion (circle); (**C**) post-fire stereotactic images of the right breast showing the position of the needle inside the suspicious lesion (circle); (**D**) 2D synthetic view of the right breast 2 days after the procedure, showing the 3mm titanium clip marking the biopsy site with neither residual microcalcifications nor hematoma; (**E**) specimen radiograph showing microcalcifications in the samples.

Once the patient is positioned with the best approach chosen in the planning phase and the breast is compressed, we perform the DBT to identify the target lesion (Figure 3A). The choice of the gauge and the aperture of the needle depend on the size, the position of the lesion, and the breast compressed thickness. In our department, we mostly use 9 G or 12 G needles with 20 mm or 12 mm apertures.

After choosing the most appropriate needle, the skin disinfection is performed. Subsequently, we administer local anesthesia. In our experience, this procedure often determines a displacement of the lesion. Thus, we acquired another DBT image to eventually retargeting it. After that, we insert biopsy needle performing the pre-fire stereotactic image with or without post-fire stereotactic images to confirm the expected needle trajectory, according to the calculated coordinates (Figure 3B,C).

A localizing post-biopsy marker clip, usually a 3 mm titanium clip, is then placed at the biopsy site, and the last image is taken to be sure that it has been released. After the needle removal, we proceed to a strong manual compression and bandaging. To evaluate the results of the procedure and the position of the clip, we prefer to perform the two-view mammography a few days after to avoid another compression that could lead to the forming or the worsening of the hematoma (Figure 3D).

When necessary, a specimen radiograph is then obtained at the of the procedure to confirm the presence of calcifications (Figure 3E).

## 3. Results

We included 85 women aged 41–84 years old (57.2 ± 7.9; mean ± standard deviation). The characteristics of the patients and of the findings are reported in Table 1.

We found 13 lesions classified as BI-RADS^®^ category R3, 32 as R4a, 23 as R4b, 16 as R4c and one as R5 at preliminary DBT. We identified 37 patients with breast cancer (56.5 ± 7.5) of which one among lesions classified as R3, six among R4a, 15 and 14 among R4b and R4c, respectively and one among R5. Thirty-seven (37) patients have benign findings (58.0 ± 8.6); of these, four were found among R4b while 12 and 21 among R3 and R4a, respectively. Finally, 11 patients (56.5 ± 6.5) have lesions classified as B3 [16]; of these, five were previously classified as R4a, four as R4b and two as R4c at preliminary DBT (Table 2). In particular, in this group of findings we diagnosed nine atypical ductal hyperplasia (ADH), a sclerosing adenosis with focal atypia and papillomatosis, and a complex lesion with florid adenosis, usual ductal hyperplasia (UDH) and columnar cell atypia (CCA).

The agreement between the histological findings after VABB and the final diagnosis after surgery was almost perfect (κ: 0.92, 95% CI: 0.76–1.08; *p*-value < 0.05) (Table 3).

The higher number of BC has been diagnosed in women with high breast density (25/37, 67.5%) (Table 4). According to the literature, we found a statistically significant inverse correlation between age and breast density (−0.3667, *p* < 0.001). Nevertheless, there was no statistically significant difference in age for dense compared to non-dense in women with breast cancer.

**Figure 4:**
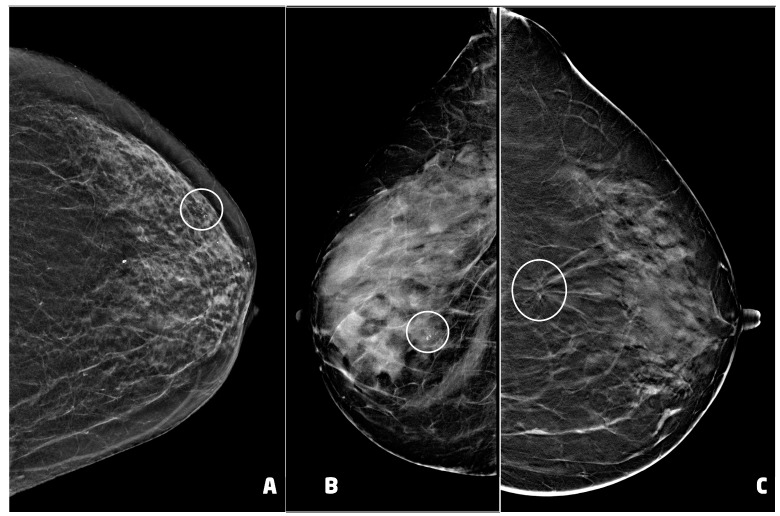
Examples of suspicious non palpable lesions undergone VABB: (**A**) 66 year-old woman, 2D synthetic view of the left breast showing a cluster of microcalcifications at the upper outer quadrant, in periareolar region (circle), ductal carcinoma in situ, G1; (**B**) 53 year-old woman, 2D-FFDM view of right breast showing a cluster of microcalcification in the equatorial region (circle), ductal carcinoma in situ, G3; (**C**) 55 year-old woman, 2D synthetic view of the left breast showing an area of architectural distortion at the union of the upper quadrants (circle), invasive ductal carcinoma, tubular variant.

Thirty (30) BC presented as microcalcifications with (four) or without (26) architectural distortion. Six (6) BC presented as architectural distortion without microcalcifications, and one BC presented as an inhomogeneous area of asymmetrical density with scattered microcalcifications (Figure 4). Of the 37 BC diagnosed, 26 were in stage 0 and 11 in stage IA (Table 1).

**Figure 5:**
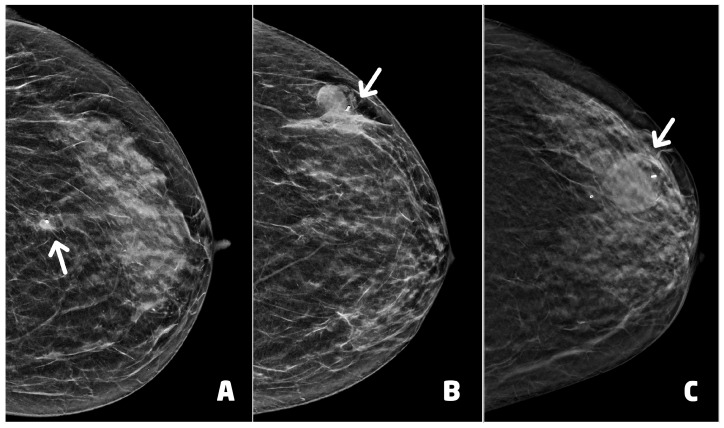
Post-procedural hematomas and clip placement (white arrows): (**A**) 55 year-old woman (see Figure 4C), clip in correct position with mild hematoma, two days after the procedure; (**B**) 62 year-old woman with lobular carcinoma in situ of the left breast, clip in correct position with moderate hematoma, two days after the procedure; (**C**) 66 year-old woman (see Figure 4A), clip in correct position with moderate hematoma.

Post-procedural hematoma was found in 12 cases (14.1%) (Figure 5). According to the classification proposed by Schaefer et al. [24], two hematomas were classified as moderate while 10 as mild. Nine hematomas were found in women who underwent VABB with a 9 G needle, while the remaining three underwent VABB with a 12 G needle.

The post-procedural clip was correctly positioned in 75 of 85 procedures (88.2%). The clip was migrated cranially in four cases, caudally in four cases and externally to lesion in the remaining two cases. One woman showed both hematoma and migration of the clip after a procedure performed with a 9 G needle.

No infection or post-procedural bleeding hemorrhage were recorded. Similarly, no procedure was repeated or cancelled.

## 4. Discussion

Breast biopsy is fundamental for the diagnosis of nonpalpable image-detected abnormalities, and the VABB technique is widely performed especially under stereotactic and DBT imaging guidance. This procedure can be performed either in the upright or prone position, depending on the department availability, the operator preference, and the lesion position.

Our department experience is based on the use of upright procedure with the digital mammography system with a 3D tomosynthesis used for the diagnostic screening, by applying an installed guidance system. The use of a DBT-guided biopsy add-on, lightweight and easily installed on a 3D mammography unit in minutes, makes biopsy sitting more ergonomic and feasible, providing multifunctionality to the room used by being able to quickly switcher between imaging units to interventional units. In addition, a vertically developing unit is more compact than a prone stereotactic biopsy machine, leaving more room for maneuver for the operating radiologist and their staff.

In clinical practice, the use of VABB-DBT is required mostly for calcifications (90%), and less commonly for spiculated masses (4.7%), asymmetric densities (2.9%), and distortions (2.4%), without US corresponding [37]. Regarding the morphology and distribution of calcifications, they are often grouped while the heterogeneous coarse type is the most commonly observed, followed by the pleomorphic, amorphous, and linear fine types [34].

After several years of practice, the procedure at our department has been simplified and made more efficient. The patient preparation is minimized to the essential. Nevertheless, although the SIR recommendations categorize percutaneous breast biopsy as having a low risk of bleeding, these do not consider the gauge of the needle used. Therefore, we consider a good indication to have anti-platelet agents discontinued when using a 9 G needle. We require the laboratory tests such as PT, PTT, INR, and platelet count analysis while in cases of patients under anticoagulant therapy we request a complete laboratory profile and conversion to seleparin therapy from one week earlier to 3 days later.

The choice of the gauge and the aperture of the needle depend on the type and deepness of the lesion as well as the characteristics of the breast. In particular, the inner diameter of the needle ranges from 11 to 7 gauge; the aperture either of 20 mm, or 12 mm. The standard biopsy device with an opening of 20 mm is commonly used for breasts with a compressed thickness of 30 mm or greater. The petite biopsy device with a smaller aperture of 12 mm, is used for thin compressed breasts (thickness less than 20 mm) and superficial lesions [15]. In our department, we mostly use 9 G or 12 G needles with 20 mm or 12 mm apertures.

Anesthesia is administered with ropivacaine 10 mg/mL. We always acquired a further DBT image after anesthesia due to the displacement of the target caused by the injection itself. Only after checking the correct position of the target do we insert biopsy needle. Usually, we performed both pre-fire and post-fire images. Depending on the biopsy needle size and target type, we obtained an average of six to 12 samples, typically extracted from even or odd clock-face positions spanning a full 360° rotation. This approach ensures adequate spacing between each vacuum-assisted sample. In cases where the target is slightly displaced from the needle, additional directional sampling may be necessary. Following this, the biopsy device is switched to wet lavage mode, and the biopsy cannula is rotated 360° to irrigate the biopsy cavity with saline, collecting any free tissue samples. Wet lavage is continued until the fluid retrieved in the tubing is clear. Subsequently, dry lavage, or continuous aspiration, can be performed to collapse the biopsy cavity and remove any remaining hematoma or saline.

As aforementioned, the risk of significant bleeding hemorrhage is about 0.1%. To reduce the bleeding, manual compression for about 5–10 min and bandaging are recommended, usually with the addition of cold ice pack, as well as the use of vitamin K, either orally, sublingually or directly at the bleeding site in cases of more prolonged bleeding. At our department, the use of the EVIVA Breast Biopsy system (Hologic, Bedford, MA, USA) with a Y-valve double-way system with transparent tubes allows us to observe the passage of the samples and the possible presence of bleeding in real time. If the latter is excessive, we inject one or two tranexamic acid 500 mg/5 ml phial in the biopsy site, during the lavage time.

In our experience, no significant bleeding hemorrhage was recorded after the procedure. Post-procedural hematoma was found in 14.1% of patients. Of these, 10 were mild and two were moderate. These results were consistent with the literature.

By the end of the procedure, a localizing post-biopsy marker clip is placed and a 2D image is acquired to verify that the clip has been released. To verify the outcome of the procedure and the correct placement of the marker, especially when there has been significant bleeding, we prefer to perform the two-view mammography a few days after the procedure to avoid further compression that could lead to the forming or the worsening of the hematoma. These additional images, usually acquired in both craniocaudal and mediolateral or lateromedial views, are fundamental to assess the final position of the clip. Moreover, they evaluate the appropriate targeting, the presence of residual calcifications or a residual lesion, and the presence of a hematoma [18].

Clip migration has been reported in up to 20% of cases [37]. In our experience, clip was migrated only in 10 patients (11.8%). Even this data was consistent with the literature.

Usually, we perform both pre-fire and post-fire images as well as we try to collect the highest number of samples, completing each step of the whole procedure, as mentioned. However, patients may exhibit poor compliance with the procedure, often due to factors such as anxiety and fear. In these cases, the procedure is streamlined as much as possible, acquiring only pre-fire images and a maximum of 6–8 samples with no post biopsy images.

In cases where psychological stress is more significant and the likelihood of syncope is particularly high, atropine 0.5 mg/1 mL is administered prior to the procedure. From 2019 to 2022, no procedure was interrupted due to the occurrence of syncope episodes. No procedure has been repeated.

The correlation of the histologic finding with the imaging of the lesion is essential. BIRADS lexicon and classification have proven to be useful in predicting the likelihood of malignancy in radiologically assessed breast lesions [38].

The question of whether the BIRADS is correctly applied can only be answered by directly correlating the histological and the imaging findings in each given case. Jörg et al. [39] found that of 232 preoperative core needle and/or vacuum-assisted breast biopsies classified as category B1 (138, 59.5%) and B2 (94, 40.5%), the re-biopsy found malignancy in a significant percentage of cases. In particular, 51 of 138 B1 cases (37%) underwent re-biopsy finding malignancy in 19 cases, and premalignant lesions in three cases. Similarly, 57 of 94 B2 cases (66%) underwent consecutive direct surgery or re-biopsy. Of these, malignancy was diagnosed histologically in 26 cases (45.6%). Consequently, it is essential to follow a standardized and scrupulous procedure because in case of a discrepancy, further intervention, such as repeat core needle biopsy or surgical biopsy or alternatively close monitoring of lesions found to be benign, is required [15]. In some cases, US-guided VAB has been shown to be a valuable alternative to open surgery for breast lesions with imaging-pathology discordance with an upgrade rate from 4.6 to 22.7% [40,41,42].

In our experience, the agreement between histopathological findings after VABB and breast surgery was almost perfect with only one case upgraded from ADH to BC and two cases evaluated as highly suspicious for malignancy confirmed as BC after surgery.

There are several data in the literature about the diagnostic accuracy and reliability of DBT-guided VABB biopsy. Penco et al. [43] reported a sensitivity of 99.7% to 100% and a false-negative rate of 1.7% to 7.1% in 4086 patients in a 10-year study. Tsai et al. [44] reported a sensitivity of 95.24%, with a false-negative rate of 4.76% and a negative predictive value (NPV) of 99.61% in 817 patients in a 5-year study. Similarly, Kettritz et al. [45] obtained sensitivities and NPVs above 99%. Bohan et al. [46] found a specificity of 100% and sensitivity of 91.3% with a PPV of 100% for malignancy and a high NPV (81.8%) for benignity but a higher false-negative rate of 20% due to smaller sample size.

On the other hand, the underestimation rate of DCIS and ADH is one of the principal issues. Tsai et al. [44] reported an underestimation rate of DCIS of 16.7%, while Kettritz et al. [45] revealed that the underestimation rate of ADH and DCIS was 24% and 12%, respectively. Badan et al. [47] reported an underestimation rate of ADH and of DCIS of 25% and 14.28%, respectively. According to Inyoung Youn et al. [48], the underestimation rate of ADH was 33.3%.

In general, the malignancy rate found in the literature ranges from 19% to 24%, justifying the use of DBT-guided VABB [49,50]. In our experience, most of our cases presented as microcalcifications and/or architectural distortions and all the diagnosis of BC were made at an early stage (0 and IA). The higher number of BC has been diagnosed in women with high breast density with a statistically significant inverse correlation between age and breast density. These results are consistent with the literature [51,52]. Nevertheless, there was no statistically significant difference in age for dense compared to non-dense in women with breast cancer. This aspect is probably due to the characteristics of the population in which the number of women with high breast density was higher than that with low breast density. Consequently, the greatest number of lesions, both benign and malignant, were found in women with dense breasts.

This study has some limitations. First, this is a single-institution study. All the mammograms were obtained from a single mammographic unit, and only the Quantra software was available in our institution to evaluate breast density. Furthermore, the evaluation of BD and the histopathological analysis were performed by radiologists and pathologists with a similar workplace but different levels of experience. This may have led to similar assessments based on shared practice patterns. Finally, the small sample size might limit the significance of the results. A broader sample, the introduction of prone-VABB, and the incorporation of additional software for breast density assessment could be the next steps to develop future studies.

## 5. Conclusions

Compared to the literature, our results confirmed the fundamental role of DBT-guided VABB in achieving a timely diagnosis for nonpalpable lesions without US correlation, offering a safe and minimally invasive approach and a high histopathological concordance, when the technique is executed correctly. In Table 5, we provide suggestions for clinical practice based on our experience.

Recently, studies suggested that it is reasonable to perform VABB as definitive treatment for certain B3 lesions (specifically classical lobular neoplasia, flat epithelial atypia, radial scar, and papillary lesions), while surgical excision should continue as the mainstay of treatment for atypical ductal hyperplasia and for all the lesions classified as B4 or B5 [53,54]. With a view to the future, thanks to the increasingly popularity of Contrast-enhanced Digital Mammography (CEDM), which compared to breast MRI is more affordable, better tolerated by patients and quicker to perform [55], the diagnostic accuracy of DBT-VABB could be improved, making it possible to discriminate in cases of several suspicious lesions those with a higher risk of malignancy and above all to facilitate patient repositioning and lesion targeting at the time of biopsy. Precisely in this perspective, VABB under CESM guidance is the most recent and promising innovation to focus on for future developments.

## Figures and Tables

**Table 1:** Main characteristics of the patients and the findings. ADH: atypical ductal hyperplasia; DCIS: ductal carcinoma in situ; CCA: columnar cell atypia; ER: estrogen receptor; IDC: invasive ductal carcinoma; ILS: invasive lobular carcinoma; LCIS: lobular carcinoma in situ; PgR: progesterone receptor; UDH: usual ductal hyperplasia.

Breast Cancers:										
Age	BIRADS	Biopsy	Diagnosis	Grade	TNM	Stage	ER	PgR	Ki67	Her2
41	B4c	B5	IDC + DCIS	G2	pT1a pN0	IA	90	90	10	1+
45	B4b	B5	IDC	G3	pT1a pN0	IA	0	0	60	3+
46	B4a	B5	DCIS	G3	pTis	0	+	/	/	/
46	B4b	B4	IDC tubular	G1	pT1a pN0	IA	90	60	10	1+
47	B4c	B5	DCIS	G1	pTis	0	40	1	5	1+
48	B4c	B5	DCIS	G1	pTis pN0	0	+	/	/	/
50	B4b	B5	DCIS	G1	pTis	0	+	/	/	/
50	B4c	B5	ILC	G1	pT1b pN0	IA	95	95	3	0
51	B4c	B5	DCIS	G1	pTis	0	-	/	/	/
51	B4b	B3	DCIS + ADH	G1	pTis	0	+	/	/	/
53	B4a	B5	DCIS	G1	pTis	0	30	10	/	/
53	B4c	B5	DCIS	G3	pTis pN0	0	80	2	20	0
53	B4b	B4	DCIS	G2	pTis	0	0	0	90	0
54	B4c	B5	IDC + DCIS	G3	pT1c pN0	IA	90	2	30	2+/0
54	B5	B5	DCIS	G2	pTis pN0	0	40	3	30	3+
54	B4b	B5	DCIS	G3	pTis	0	0	0	75	0
55	B4b	B5	IDC tubular	G1	pT1a pN0	IA	70	60	10	0
56	B4a	B5	CDIS	G1	pTis	0	+	/	/	/
56	B4c	B5	IDC NST + DCIS	G1	pT1c pN0	IA	90	70	10	3
56	B4c	B5	DCIS	G3	pTis pN0	0	0	0	/	/
56	B4b	B5	IDC	G2	pT1b pN0	IA	90	90	15	0
57	B4a	B5	DCIS + ADH	G1	pTis pNx	0	80	10	/	/
57	B4b	B5	DCIS multifocal	G3	pTis pN0	0	0	0	50	3+
58	B4a	B5	DCIS	G3	pTis pN0	0	0	0	20	3+
60	B4b	B5	DCIS	G3	pTis	0	90	2	10	2+
60	B4c	B5	DCIS	G3	pTis pN0	0	+	/	/	/
62	B4a	B5	LCIS	G2	pLis	0	90	/	/	/
62	B4b	B5	DCIS	G2	pTis pN0	0	100	100	/	/
62	B4c	B5	DCIS + ADH	G1	pTis pNx	0	+	/	/	/
62	B4b	B5	DCIS	G1	pTis pNx	0	80	80	2	1+
63	B4b	B5	DCIS	G1	pTis pN0	0	70	70	5	2+
66	B4c	B5	DCIS	G3	pTis pN0	0	80	10	/	/
66	B3	B5	DCIS	G1	pTis pNx	0	90	20	10	1+
69	B4b	B5	DCIS	G3	pT1mic pN0	IA	90	80	25	1+
70	B4c	B5	ILC	G2	pT1c pN1mi	IA	70	80	25	0
70	B4b	B5	DCIS	G3	pTis	0	+	/	/	/
72	B4c	B5	DCIS	G3	pT1mi pN0	IA	80	20	20	2+
B3 lesions after surgery:										
49	B4a	B3	Sclerosing adenosis with CCA and ADH							
49	B4a	B3	UDH and ADH							
50	B4a	B3	Florid adenosis, UDH and CCA							
53	B4b	B3	ADH							
53	B4a	B3	ADH with UDH							
56	B4c	B3	ADH							
58	B4c	B3	Radial scar with atypia and papilloma							
59	B4a	B3	ADH							
59	B4b	B3	ADH							
67	B4b	B3	UDH with ADH							
69	B4b	B3	ADH							

Benign findings are mainly represented by the following lesions: sclerosing adenosis without atypia, UDH, apocrine metaplasia and fibrosis.

**Table 2:** Classification and relationship of findings after mammography, VABB and surgery. BC: breast cancer; DBT: digital breast tomosynthesis; VABB: vacuum-assisted breast biopsy. BI-RADS classification performed according to 5th Edition. Age: mean ± standard deviation.

Type of Exam	Classification of the Findings					Total	Age	Median
Mammography with DBT								
	R3	R4a	R4b	R4c	R5			
	13	32	23	16	1	85	57.2 ± 7.9	56
VABB								
B5	-	6	13	14	1	34	57.1 ± 7.5	56
B4	-	-	2	-	-	2	49.5 ± 3.5	49.5
B3	1 *	5	4	2	-	12	56.5 ± 6.5	56
B2	12	21	4	-	-	37	58.0 ± 8.6	57
Surgery								
BC	1 *	6	15	14	1	37	56.5 ± 7.5	56

* This patient, with findings classified as BI-RADS category 3 at DBT and as B3 at VABB, underwent surgery with a diagnosis of malignancy.

**Table 3:** Agreement between the histological findings after VABB and the final diagnosis after surgery, according to the interpretation of weighted kappa values provided by Landis J.R. and Koch G.G. (κ: 0.92 = almost perfect agreement).

	Surgery				
VABB	Benign Findings	B3 Lesions	B4 Lesions	BC	TOT
B2	36	0	0	0	36
B3	1	11	0	1	13
B4	0	0	0	2	2
B5	0	0	0	34	34
Total	37	11	0	37	85

Agreement: κ = 0.92, standard error: 0.08; 95% CI: 0.76–1.08; *p*-value < 0.05.

**Table 4:** Diagnosis by age and breast density (BD). Age: mean ± standard deviation.

Diagnosis	BD	N.	Age	BD Category	N.	Age
BC (37)	B	12	59.2 ± 7.7	Non-dense	12	59.2 ± 7.7
	C	19	57.2 ± 5.6	Dense	25	55 ± 7.1
	D	6	53.2 ± 7.6			
B3 lesions (11)	B	4	61.2 ± 6.9	Non-dense	4	61.2 ± 6.9
	C	7	53.9 ± 4.4	Dense	7	53.9 ± 4.4
Benign (37)	A	1	64	Non-dense	15	60.8 ± 7.5
	B	14	60.6 ± 7.7			
	C	19	57.3 ± 8.9	Dense	22	56.1 ± 8.9
	D	3	48.7 ± 1.2			

**Table 5:** Guidelines for clinical practice. Procedure suggested for DBT-guided vacuum-assisted breast biopsy (VABB). DBT: digital breast tomosynthesis; CC: cranio-caudal projection; INR: international normalized ratio; ML: medio-lateral projection; LM: latero-medial projection; PT: prothrombin time; PTT: partial thromboplastin time.

Guidelines for Clinical Practice	
Before VABB	
(1)	Clinical and familiar history (with particular attention to anticoagulant therapy and allergy to anesthetic drugs)
(2)	Evaluation of laboratory tests (PT, PTT, INR, platelet count)
(3)	DBT images evaluation (to choose the correct gauge and aperture of the needle)
VABB examination	
(4)	Full explanations of the procedure (written informed consent; is there psychological stress?)
(5)	DBT-VABB procedure a. positioning images b. post-anesthesia images c. pre-fire images d. post-fire images (optional if poor compliance) e. post-clip images (optional if poor compliance)
(6)	Bandage with compression
(7)	Patient monitoring (at least 10 min)
After VABB	
(8)	Post-procedure DBT images (LM/ML and CC projections) to evaluate the whole procedure and the correct clip position 2–3 days after the procedure

## Data Availability

The dataset can be found on saniarp.it, the Caserta LHA reporting database, and from the register of our daily activities.

## Supplementary Materials

The following supporting information can be downloaded at: https://www.mdpi.com/article/10.3390/cancers15245720/s1, Table S1: STROBE Statement—checklist of items that should be included in reports of observational studies.
